# Prenatal detection of Gorlin–Goltz syndrome: a case report and focused review of the literature

**DOI:** 10.3389/fmed.2026.1659100

**Published:** 2026-04-01

**Authors:** Kathrin Oelmeier, Judit Horváth, Rebekka Vogtmann, Matthias Meyer-Wittkopf, Mareike Möllers, Ralf Schmitz, Kathleen Marie Oberste, Daniela Willy

**Affiliations:** 1 Department of Gynecology and Obstetrics, University Hospital Münster, Münster, Germany; 2 Institute of Human Genetics, University Hospital of Münster, Münster, Germany; 3 Department of Prenatal Diagnosis, Mathias-Spital Rheine, Rheine, Germany; 4 Department of Gynecology and Obstetrics, Florence-Nightingale-Krankenhaus, Düsseldorf, Germany

**Keywords:** case report, Gorlin-Goltz syndrome, NBCCS, Nevoid basal cell carcinoma syndrome, prenatal ultrasound, *PTCH1*

## Abstract

**Introduction:**

Gorlin–Goltz syndrome is a rare tumor-predisposing condition caused by genetic variants in the *PTCH1* and SUFU pathways. The list of genetic variants associated with Gorlin–Goltz syndrome is not yet exhaustive. In this study, we present a unique case with typical features and a genetic variant previously classified as a variant of uncertain significance.

**Case presentation:**

In this case, genetic testing was performed due to fetal hydronephrosis and omphalocele. Additional features included macrocephaly with hypertelorism and agenesis of the corpus callosum. Genetic testing revealed a heterozygous *de novo* splice-region variant, c.945 + 5G > T p.?, in *PTCH1* (NM_000264.5), predicted to result in an in-frame deletion of the last 87 base pairs of exon 6.

**Discussion:**

In the presence of typical prenatal features, this genetic variant was reclassified as likely pathogenic for Gorlin–Goltz syndrome. As prenatal features are often non-specific, and hydronephrosis and omphalocele are relatively common, Gorlin–Goltz syndrome should be included in the differential diagnosis during genetic testing for these fetal anomalies, particularly when additional syndromic features are present. Close interdisciplinary teamwork between prenatal ultrasound specialists and geneticists is essential to correctly evaluate non-specific fetal abnormalities and establish an accurate diagnosis in the fetus. Sometimes, as in this case, this process can lead to the reclassification of known genetic variants or even the identification of novel variants.

## Introduction

Gorlin–Goltz syndrome is a rare tumor-predisposing condition (1). It is also referred to as Nevoid basal cell carcinoma syndrome (NBCCS), and the clinical features of the syndrome were first described by Gorlin and Goltz in 1960 (2). The incidence of Gorlin–Goltz syndrome is estimated to range between 1:30827 and 1:256000 (3, 4).

Inheritance follows an autosomal dominant pattern with a high degree of penetrance and variable expressivity. In approximately 60% of cases, pathogenic variants in the patched gene 1 (*PTCH1*) are responsible for Gorlin–Goltz syndrome, while an additional 4% of cases are attributable to Suppressor of Fused Homolog (SUFU) pathogenic variants. In the remaining 36% of cases, no pathogenetic variant is identified (5).

Postnatal features of Gorlin–Goltz syndrome include a characteristic appearance with macrocephaly, hypertelorism, frontal bossing, and facial milia. Additional manifestations comprise recurrent odontogenic jaw keratocysts and ectopic calcifications of the falx cerebri or the hands and feet. Although life expectancy is generally not reduced, the risks of basal cell carcinomas and medulloblastomas are markedly increased, and patients are typically enrolled in intensive surveillance programs. Benign tumors, such as cardiac or ovarian fibromas, are also relatively common. Neurologic impairment is uncommon but may occur in severe cases (6).

As with many rare disorders, Gorlin–Goltz syndrome is frequently underdiagnosed postnatally, and a set of clinical diagnostic criteria has therefore been established (6). The postnatal diagnostic criteria are summarized in Table 1. Prenatal diagnosis is even more challenging because Gorlin–Goltz syndrome is a rare condition with more than 100 clinical features that have been described, many of which are non-specific (7). Consequently, no formal prenatal diagnostic criteria have yet been defined. Reported prenatal manifestations include facial anomalies, hydrocephalus, macrocephaly, and chylothorax (7, 8). However, these findings may also occur in more common genetic syndromes, such as trisomy 13 or trisomy 18, complicating differential diagnosis.

**Table 1:** Diagnostic criteria for postnatal diagnosis of Gorlin–Goltz syndrome.

Major criteria
More than two basal cell carcinomas (BCC) or one BCC under the age of 20
Odontogenic keratocysts of the jaw (histopathologically proven)
Three or more palmar or plantar pits
Bilamellar calcification of the falx cerebri
Bifid, fused, or markedly splayed ribs
First degree relative with Gorlin-Goltz syndrome
Minor criteria
Macrocephaly determined after adjustment for height
Congenital malformations: cleft lip or palate, frontal bossing, coarse face, moderate or severe hypertelorism
Other skeletal abnormalities: Sprengel deformity, marked pectus deformity, marked syndactyly of the digits
Radiological abnormalities: bridging of the sella turcica, vertebral anomalies such as hemivertebrae, fusion or elongation of the vertebral bodies, modeling defects of the hands and feet, or flame-shaped lucencies of the hands or feet
Ovarian fibroma
Medulloblastoma

Two major criteria or one major criterion and two minor criteria are needed for a diagnosis of Gorlin–Goltz syndrome (6).

Given the limited number of reported postnatal cases, the spectrum of genetic variants associated with Gorlin–Goltz syndrome remains incomplete. In this unique report, we describe a fetus presenting with non-specific prenatal abnormalities on the first-trimester screening. Initial genetic testing identified a variant of uncertain significance (VUS) without any abnormalities. As pregnancy progressed, typical features of Gorlin–Goltz syndrome became apparent in the ultrasound examination, and subsequent genetic testing, combined with close interdisciplinary collaboration, enabled the reclassification ofthe variant as likely pathogenic for Gorlin-Goltz syndrome. To our knowledge, this case represents the first reported prenatal diagnosis of Gorlin–Goltz syndrome in the absence of any family members with a history of the disorder.

## Case description

The publication of this case report complies with guidelines for human studies and adheres to the World Medical Association’s Declaration of Helsinki. The patient and her partner provided written informed consent for the publication of their case, including accompanying images. Prior to submission, the participants were provided a copy of the manuscript and provided final approval for publication.

**Figure 1:**
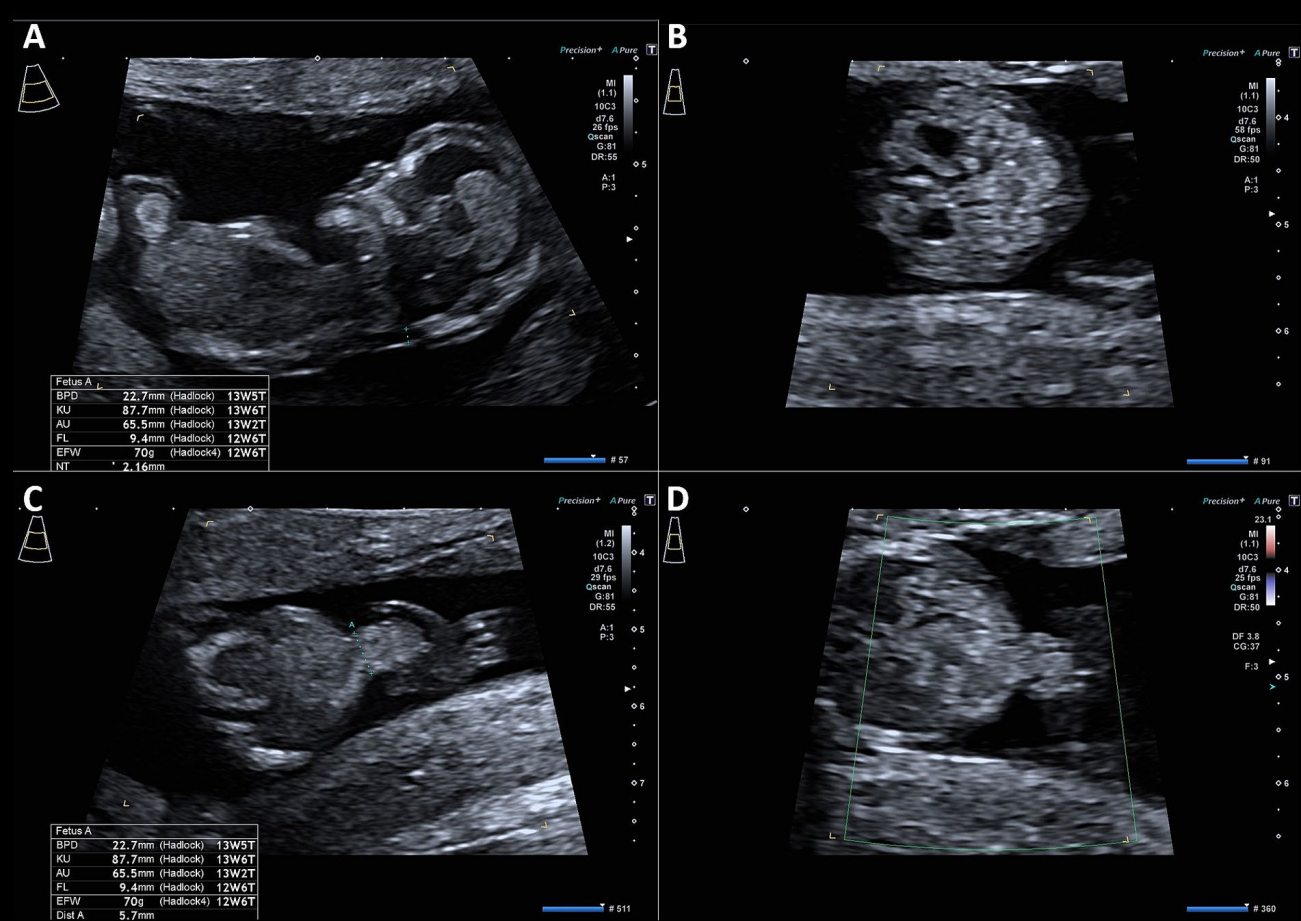
Ultrasound images of first-trimester screening showing the measurement of nuchal translucency **(A)**, bilateral hydronephrosis **(B)**, and omphalocele **(C,D)**.

A 35-year-old patient with no preexisting medical conditions was referred for prenatal ultrasound at 14 weeks of gestation due to suspected omphalocele. This was the patient’s fifth pregnancy, with a history of one term delivery, one preterm delivery at 32 weeks of gestation, and two first-trimester abortions. The child delivered at 32 weeks of gestation has cerebral palsy; otherwise, no other diseases, particularly genetic disorders, were reported in the family’s history. During the first-trimester screening at a prenatal diagnostics department, a small omphalocele containing bowel loops was confirmed. Additional findings included an umbilical cord cyst and bilateral pyelectasis. Ultrasound images from the first-trimester screening are shown in Figure 1.

**Figure 2:**
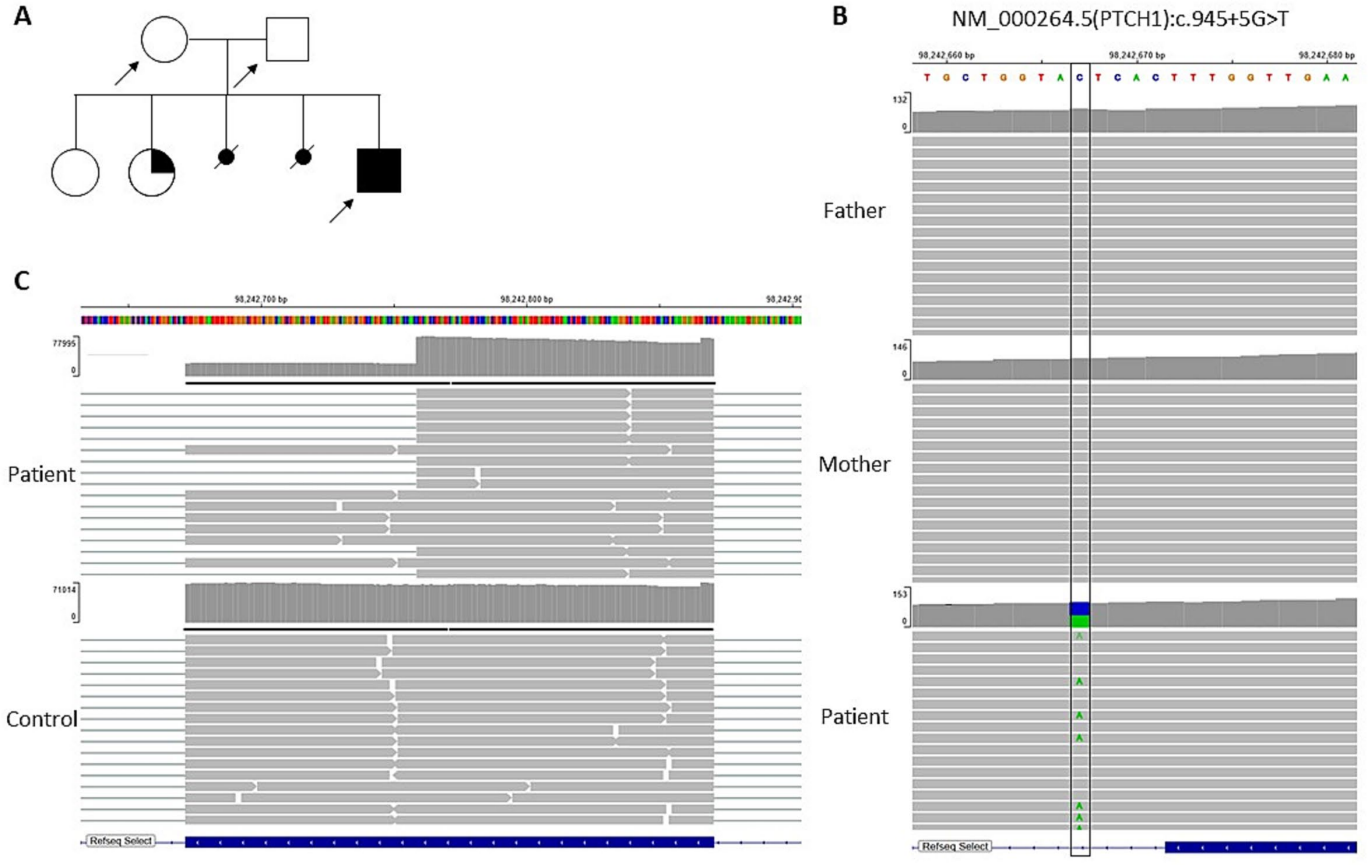
Family pedigree with arrows indicating the patient and the parents **(A)**, trio-exome analysis of the patient and his parents **(B,C)**. The integrative genomic viewer (IGV) demonstrates the *de novo* heterozygous variant c.945 + 5G > T in *PTCH1* (NM_000264.5); reference base of coding sequence shown in blue and altered base shown in green **(B)**. IGV screenshot of RNA coverage of exon 6 of *PTCH1* **(C)**.

After informed consent, a chorionic villous sampling (CVS) was performed. Genetic testing included chromosome analysis, single-nucleotide polymorphism (SNP) array analysis, multiplex ligation-dependent probe amplification (MLPA) analysis, including testing for Beckwith–Wiedemann syndrome, and trio-exome sequencing. A family pedigree was constructed and is shown in Figure 2A. Chromosome analysis, SNP array analysis, and MLPA analysis did not reveal any variants explaining the abnormal ultrasound findings in the fetus. Using exome-based trio analysis, a heterozygous *de novo* splice-region variant c.945 + 5G > T p.? in *PTCH1* (NM_000264.5) was detected in the fetus (Figure 2B). This variant was initially classified as a variant of uncertain significance (VUS) (PS2_sup, PM2_sup, PP3_sup) according to the guidelines of the American College of Medical Genetics (ACMG) and Journal Pre-proof 8 Genomics (9). For further characterization, RNA analysis revealed an in-frame deletion of the last 87 base pairs of exon 6 in *PTCH1* in 67.6% of all amplified transcripts (Figure 2C).

**Figure 3:**
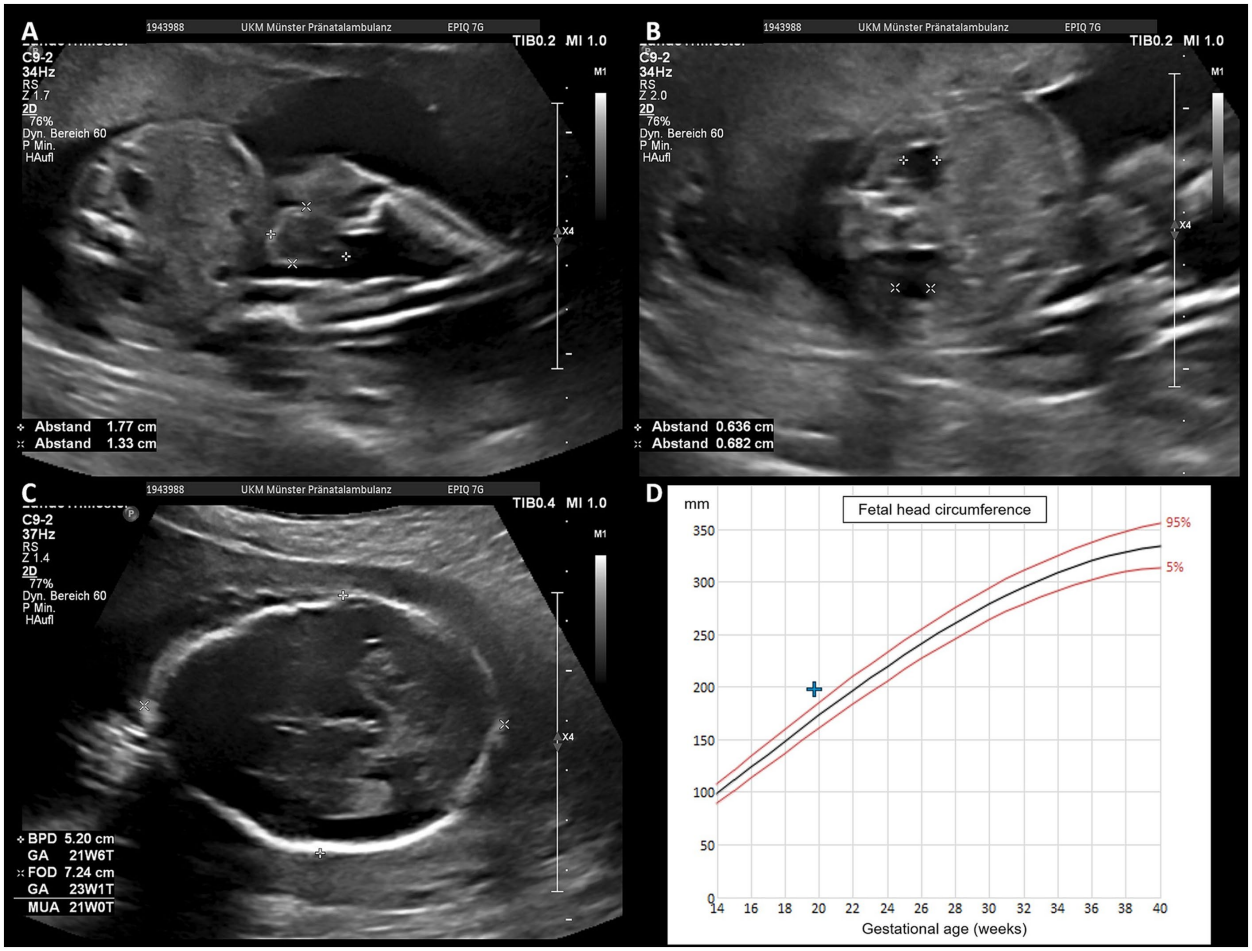
Ultrasound images and percentile curve of fetal head circumference at 20 weeks of gestation, showing umbilical cord cyst **(A)**, bilateral hydronephrosis **(B)**, and macrocephaly, along with non-visualization of cavum septum pellucidum **(C)**. **(D)** Displays the measurement of the fetal head circumference > 95th percentile.

In the meantime, the patient and her husband visited the genetics department for genetic counseling at 19 + 0 weeks of gestation and were referred to our tertiary obstetric center. At 19 + 5 weeks of gestation, a detailed prenatal ultrasound examination was performed. The fetal omphalocele, umbilical cord cyst, and bilateral pyelectasis were confirmed; additional findings included macrocephaly, hypertelorism, and agenesis of the corpus callosum (see Figure 3).

**Figure 4:**
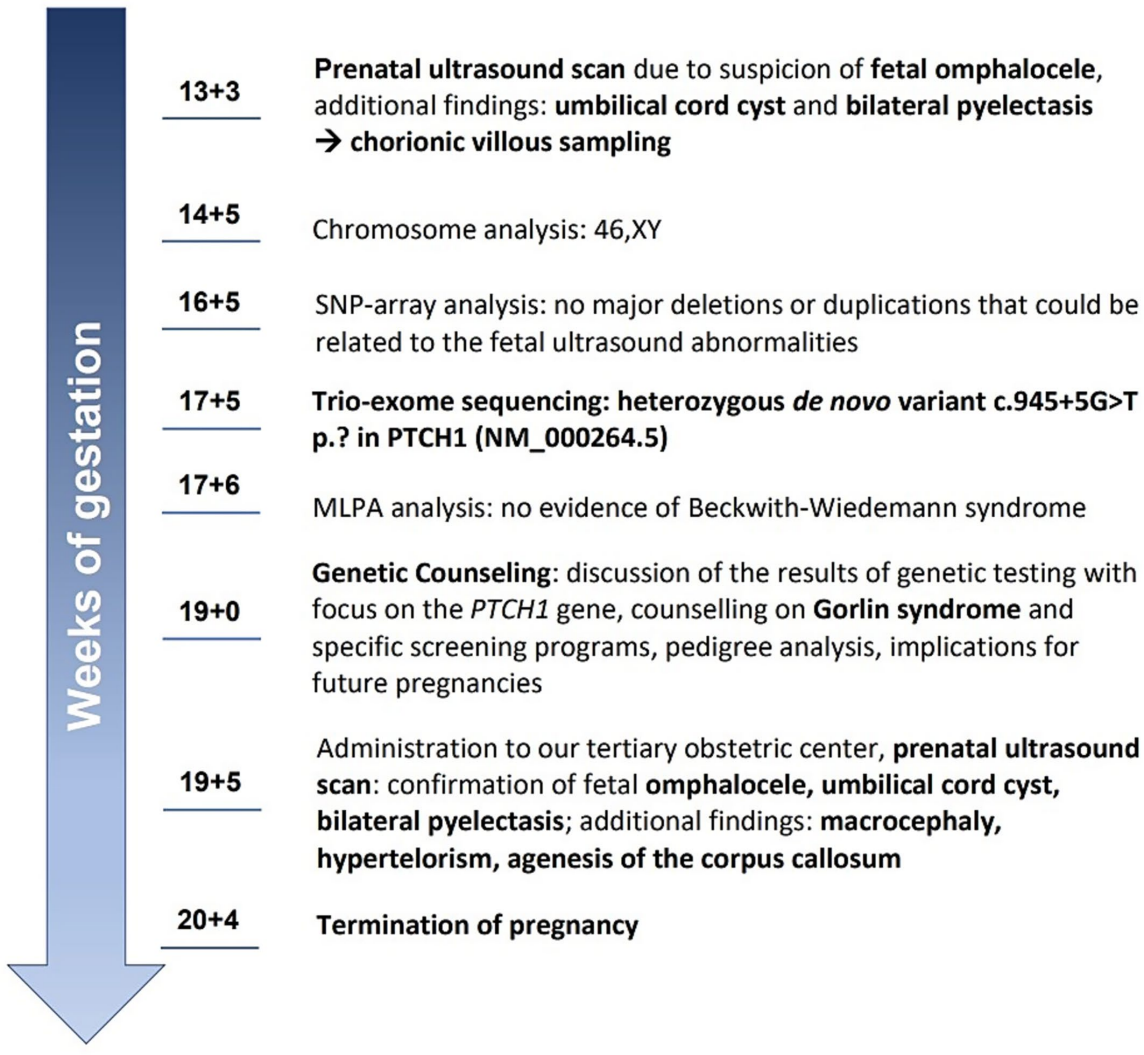
Timeline of the patient’s course.

Considering the additional ultrasound findings consistent with typical features of Gorlin–Goltz syndrome, along with the confirmed negative splice effect of the *de novo* variant c.945 + 5G > T p.? in *PTCH1*, this variant was reclassified as likely pathogenic (ACMG: PS2_MOD, PS3_SUP, PM2, PP3) and was considered responsible for the fetal abnormalities. After interdisciplinary counseling, the patient opted for termination of pregnancy (TOP) at 21 weeks of gestation. TOP was conducted without complications. Postnatal radiography of the fetus did not reveal any skeletal abnormalities. For an overview and better understanding of the patient’s course, see the timeline in Figure 4.

## Discussion and review of the literature

This case illustrates how reclassification of a genetic variant from VUS to likely pathogenic can be achieved through close collaboration between prenatal ultrasound specialists and geneticists. Initially, the detected variant was classified as a VUS. Serial ultrasound follow-ups and complementary RNA analysis were performed to enable a more robust assessment of the variant. We specifically searched for features typical of *PTCH1*-associated alterations. The sonographic abnormalities first documented on follow-up (macrocephaly, hypertelorism, and agenesis of the corpus callosum), together with abnormal RNA findings, ultimately enabled the reclassification of the variant as likely pathogenic (ACMG: PS2_MOD, PS3_SUP, PM2, PP3). Loss-of-function of *PTCH1* is a common cause of Gorlin–Goltz syndrome. Various genetic variations are known to be involved in disease pathogenesis. The majority of the known pathogenic variants in Gorlin–Goltz syndrome are single-nucleotide variants resulting in nonsense or frameshift mutations, followed by splice-site variants, such as the splice-region variant described in the present case report (10, 11). To date, only Kato et al. have reported the same sequence change and splice effect in a postnatal case presentation of Gorlin–Goltz syndrome. The affected patient presented with macrocephaly and a bifid rib at the age of 5 years (12).

One limitation of our report is that it includes only a single case. Since Gorlin–Goltz syndrome is a rare condition, there is only a limited number of case reports available. The largest case series to date describes three postnatal patients and their management of basal cell carcinomas (13). Most case reports published describe patients with Gorlin–Goltz syndrome postnatally, and there is a paucity of information on the prenatal manifestations, which partly explains the fact that rare diseases such as Gorlin–Goltz syndrome often evade prenatal detection. Among reported prenatal cases, the most common prenatal features are macrocephaly and ventriculomegaly, detected in the second trimester (14). In other cases, a positive family history prompted invasive testing and prenatal diagnosis of Gorlin–Goltz syndrome (8, 15, 16). An overview of prenatally reported cases of Gorlin–Goltz syndrome is shown in Table 2. To our knowledge, this is the first report of prenatal detection of Gorlin–Goltz syndrome in a fetus without a relevant family history.

**Table 2:** Prenatal cases of Gorlin–Goltz syndrome reported in other studies.

Author	Year	Family history	Prenatal diagnosis	Prenatal ultrasound features
Bialer et al. (8)	1994	Yes	Yes	Unilateral cleft lip, cleft palate, hydrocephalus
Hogge et al. (15)	1994	Yes	No	Macrocephaly, mild ventriculomegaly
Petrikovsky et al. (16)	1996	Yes	No	Unilateral cleft lip, frontal bossing, ventriculomegaly, macrocephaly
Geneviève et al. (7)	2005	Yes, but not known before pregnancy	No	Cardiac tumour, hydrops, mild ventriculomegaly, hydramnios

The case reported by Bialer et al. (8) is the only one in which the diagnosis was confirmed prenatally.

Against this backdrop, reporting each case of Gorlin–Goltz syndrome is essential to raise awareness and improve prenatal recognition. Since rare diseases such as Gorlin–Goltz syndrome often require genetic testing beyond conventional karyotyping, geneticists increasingly rely on the detailed prenatal ultrasound findings to interpret molecular results accurately.

In the present case, the fetus exhibited multiple soft markers such as an umbilical cord cyst and a mild bilateral pyelectasis as early as the first trimester. Differential diagnoses for these findings include chromosomal anomalies, such as trisomy 13 or trisomy 18, VACTERL association, and rare genetic diseases, such as DiGeorge syndrome or Prader–Willi syndrome (17, 18). Soft markers are defined as “minor ultrasound findings identified in the midtrimester of pregnancy that most commonly do not represent a structural abnormality and may be normal variants but are noteworthy because of their association with an increased aneuploidy risk” by the Society for Maternal-Fetal Medicine (19). Originally established to improve detection of trisomy 21 in a high-risk population, it is currently known that multiple soft markers increase the overall risk of genetic disorders, explaining the presence of several soft markers warranting detailed ultrasound evaluation and counseling regarding genetic testing (20).

Soft markers were initially evaluated for mid-pregnancy ultrasound, although some of them can already be detected during first-trimester ultrasound screening (21). While early identification of fetal abnormalities is of great importance, especially for rare diseases, typical ultrasound features may only appear later in pregnancy. Therefore, pregnancies with fetal soft markers and a normal karyotype or inconclusive genetic results should undergo ultrasound follow-up by a specialist to guide further genetic testing and facilitate the detection of even rare fetal conditions. A close collaboration between prenatal ultrasound experts and geneticists is mandatory in these cases to consider all possibilities and ensure timely diagnosis. Genetic testing has taken great strides over the past decade. Especially, whole-exome sequencing (WES) enables the detection of a broad spectrum of variants in fetuses with detected abnormalities and a normal karyotype (22). The annotation and interpretation of WES data remains challenging due to the large amount of data generated by this technique. As demonstrated in this case, detailed information on fetal anatomy and close collaboration with experts in prenatal ultrasound can help geneticists interpret WES data to make a swift and accurate diagnosis, especially in rare diseases.

## Data Availability

The datasets presented in this study can be found in online repositories. The names of the repository/repositories and accession number(s) can be found in the article/supplementary material.
